# All You Need Is Fats—for Seizure Control: Using Amoeba to Advance Epilepsy Research

**DOI:** 10.3389/fncel.2018.00199

**Published:** 2018-07-11

**Authors:** Eleanor C. Warren, Matthew C. Walker, Robin S. B. Williams

**Affiliations:** ^1^Centre for Biomedical Sciences, School of Biological Sciences, Royal Holloway, University of London, Egham, United Kingdom; ^2^Department of Clinical and Experimental Epilepsy, Institute of Neurology, University College London, London, United Kingdom

**Keywords:** decanoic acid, *Dictyostelium*, epilepsy, ketogenic diet, ketones, non-animal models

## Abstract

Since the original report of seizure control through starvation in the 1920s, the ketogenic diet has been considered an energy-related therapy. The diet was assumed to be functioning through the effect of reduced carbohydrate intake regulating cellular energy state, thus giving rise to seizure control. From this assumption, the generation of ketones during starvation provided an attractive mechanism for this altered energy state; however, many years of research has sought and largely failed to correlate seizure control and ketone levels. Due to this focus on ketones, few studies have examined a role for free fatty acids, as metabolic intermediates between the triglycerides provided in the diet and ketones, in seizure control. Recent discoveries have now suggested that the medium-chain fats, delivered through the medium-chain triglyceride (MCT) ketogenic diet, may provide a key therapeutic mechanism of the diet in seizure control. Here we describe an unusual pathway leading to this discovery, beginning with the use of a tractable non-animal model—*Dictyostelium*, through to the demonstration that medium-chain fats play a direct role in seizure control, and finally the identification of a mechanism of action of these fats and related congeners leading to reduced neural excitability and seizure control.

## Introduction

Identifying the key therapeutic target of drugs is of great importance for biomedical science, since this enables rapid screening to develop improved compounds (Chang et al., 2012), and strengthens our understanding of the basic physiology underlying disease phenotypes. To confirm a mechanism of a compound it is essential to remove or silence the target gene, in order to demonstrate a subsequent loss of response to the compound. However, ablation of potential protein targets in mammalian models is problematic, due to the diploid nature of cells (making gene ablation difficult) and the complex array of related proteins often with overlapping catalytic function (e.g., in various isoforms or protein families). To address these issues, simple tractable models can be used, where gene ablation is rapid and efficient and a low complexity genome provides less redundancy in cellular function (Williams et al., 2002), to provide innovative proposals relating to drug targets that can then be validated in mammalian models. Using this approach, cells lacking the proposed target, having lost response to the compound, would confirm a direct activity for the compound against the target.

## An Innovative Tractable Model System Provides the Great Leap Forward

**Figure 1:**
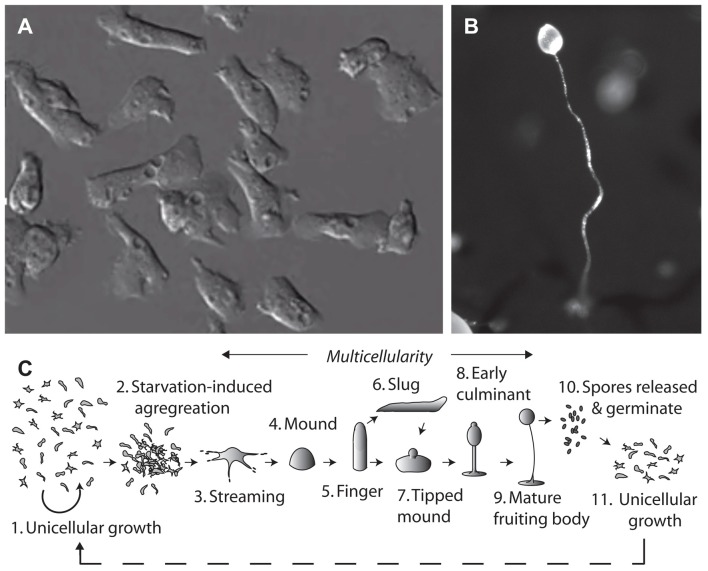
The tractable model organism, *Dictyostelium discoideum* has been used to investigate cellular mechanisms of epilepsy treatments and medium-chain triglyceride (MCT) ketogenic diet associated fatty acids. The organism can exist in **(A)** a single cell stage, with cells around 10 μm in diameter. Due to their haploid nature, genes can be easily ablated and isogenic mutants analyzed in drug target studies. Under starvation conditions *Dictyostelium* cells form **(B)** multicellular fruiting bodies, of around 1–2 mm in height, comprising a spore head held above the substratum by dead vacuolated stalk cells, where the process of aggregation and development has been widely studied. **(C)** The life cycle of *Dictyostelium* begins with unicellular growth, where cells consume bacteria and divide by binary fission. Following the onset of starvation, cells enter a development cycle where cells aggregate and enter a multicellular stage through streaming to form a mound and then finger structure. Motile slugs are able to migrate towards favorable locations and develop, or development may progress straight into a tipped mound, early culminant and then mature fruiting body. Spores within the head of the fruiting body, which are able to survive unfavorable conditions, can then be released, and germinate to re-enable unicellular growth.

The social amoeba, *Dictyostelium discoideum*, provides an unconventional system for molecular neuroscience research. This organism grows naturally in the leaf litter of temperate forests, existing in both single and multicellular stages (Figure 1; Williams et al., 2006). *Dictyostelium* belongs to the Phylum Amoebozoa, where phylogenetic analysis suggests that it diverged from the animal linage after plants, but before yeast and fungi. Despite the earlier divergence, many *Dictyostelium* proteins maintain more homology with human proteins than those of unicellular fungi (Eichinger et al., 2005). The haploid nature of *Dictyostelium* enables rapid gene ablation by insertional mutagenesis (Faix et al., 2013), and the production of mutant libraries (Kuspa, 2006). The model can then be used to investigate acute cellular effects, chronic growth effects and developmental effects of compounds to demonstrate cellular function (Robery et al., 2013; Waheed et al., 2014; Cocorocchio et al., 2016, 2018). It is important to verify that targets identified from mutant library screens are specific to the compound of interest and are not conferring broad resistance to a range of compounds. Furthermore, while *Dictyostelium* contains many proteins highly conserved with humans, some proteins are absent or have non-conserved functions. It is therefore not possible to make translatable findings about non-conserved proteins in *Dictyostelium*, and due to potential differences in cellular function of *Dictyostelium* and human proteins, it is imperative to validate findings in mammalian models.

*Dictyostelium* has been used in a range of projects to investigate the cellular and molecular mechanisms of the widely used epilepsy treatment valproic acid (VPA). It has been used to demonstrate a common mechanism of action of bipolar disorder treatments through inositol depletion (Williams et al., 2002; Eickholt et al., 2005), and in a conserved mechanism to regulate the MAP Kinase pathway in neuroprotection (Boeckeler et al., 2006). *Dictyostelium* has been valuable in identifying an uptake mechanism for VPA via an orthologe of the mammalian solute carrier family 4 (SLC4) bicarbonate transporter (Terbach et al., 2011). Importantly, it was also the first system to suggest a mechanism of VPA in regulating phosphoinositide turnover in relation to seizure control (Xu et al., 2007; Chang et al., 2012), which was subsequently validated using *in vivo* mammalian models (Chang et al., 2014). *Dictyostelium* was also employed in the identification of a range of compounds related to VPA that showed efficacy in neuroprotection and seizure control in mammalian models (Chang et al., 2013, 2015, 2016). One of the compounds identified through this pathway, decanoic acid, provides a major constituent administered in the medium-chain triglyceride (MCT) ketogenic diet.

## Metabolism of the Ketogenic Diet

Ketogenic diets have been used to treat seizures since the 1920s (Wheless, 2008). A modified form of the diet, the MCT ketogenic diet introduced in 1971 (Neal, 2017), provides a restricted carbohydrate diet, with around 45% of dietary energy delivered as fatty acids, an improvement on the classic ketogenic diet which makes up 60%–80% of dietary energy. This advancement of the diet allows more carbohydrate and protein to be consumed which improves tolerability and reduces gastrointestinal side effects. While the classic ketogenic diet relies on long-chain fatty acids, the MCT ketogenic diet provides energy in the form of medium-chain fatty acids within triglycerides, consisting of the eight-carbon octanoic acid and the ten-carbon decanoic acid in a 40–60 ratio (Sills et al., 1986; Liu, 2008). Cleavage of triglycerides in the gut leads to the release of free fatty acids, which are absorbed through the gut wall and are metabolized in the liver, where β-oxidation leads to the production of ketone bodies (β-hydroxybutyrate, acetoacetate and acetone) which are only seen in patients on a carbohydrate restricted (or starvation) diet (Haidukewych et al., 1982; Augustin et al., 2018). Although most fatty acids are degraded at this stage (Sills et al., 1986), some medium-chain fatty acids along with ketone bodies are distributed via the vascular system throughout the body and to the brain (Wlaź et al., 2012, 2015).

## Seizure Control Activities of Ketones and Free Fatty Acids

**Figure 2:**
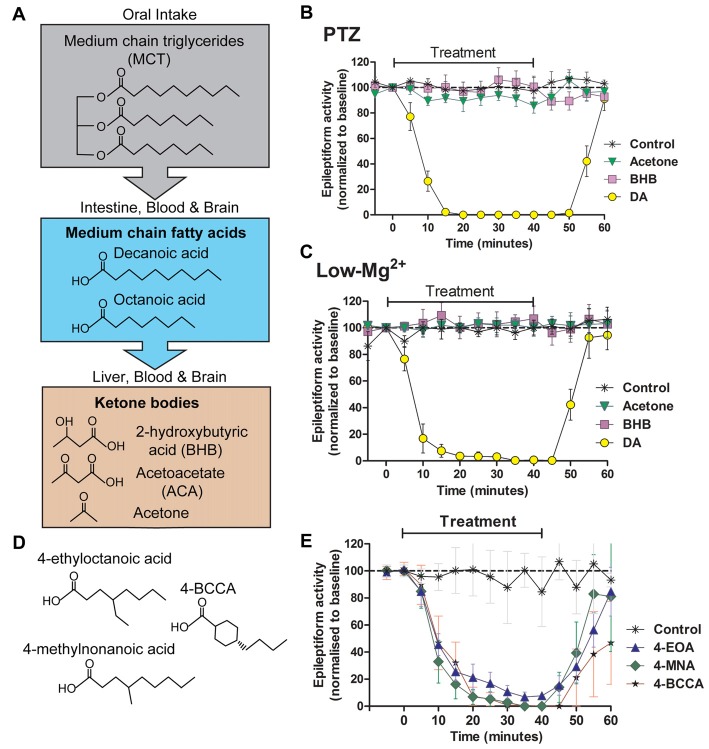
Seizure-like activity in an *in vivo* model is acutely blocked by decanoic acid (DA) and related compounds but not by ketones. The MCT ketogenic diet involves the oral intake of **(A)** medium-chain triglycerides, which are converted into the fatty acids decanoic acid and octanoic acid in the intestine. These medium-chain fatty acids are then transferred to the liver, where they are further metabolized to form ketone bodies. Fatty acids and ketones are transported in the blood to the brain where they are able to cross the blood brain barrier. Following the identification of decanoic acid as a potential therapeutic effector of the MCT ketogenic diet in *Dictyostelium*, its seizure control activity was compared to that of the ketones acetone and β-hydroxybutyrate (BHB), with seizure-like activity induced in a rat hippocampal slice model following **(B)** pentelentetrazol (PTZ) or **(C)** low magnesium treatment. In both models, epileptiform activity was not blocked by either ketones (BHB or acetone) at high concentrations (10 mM). In contrast, the medium-chain fatty acid, DA rapidly blocked activity at 1 mM. Data is derived from Chang et al. (2016). **(D,E)** A range of novel compounds and related structures implicated through the use of *Dictyostelium* have also been demonstrated to show seizure control activity, where seizure-like activity is induced in a rat hippocampal slice model following PTZ treatment. Data derived from Chang et al. (2014).

As the most investigated mechanism for ketogenic-diet dependent seizure control, many studies have focused on a role of ketones, with variable outcomes. In the absence of dietary carbohydrates, ketones are generated and distributed at concentrations of around 5 mM (Veech, 2004), where they are thought to provide an alternate energy source to glucose. In clinical studies, ketone levels poorly correlate with anticonvulsant efficacy, and this ketone-based mechanism has not been widely supported in animal model studies (Likhodii et al., 2000; Thavendiranathan et al., 2000). Ketones have been demonstrated to regulate GABA and glutamate levels (Lutas and Yellen, 2013), but do not directly act at GABA or glutamate receptors at physiological concentrations (Donevan et al., 2003), nor do they directly alter hippocampal synaptic transmission (Thio et al., 2000). Ketones do not directly block seizure activity in hippocampal slice models, induced to generate seizure-like activity with either pentelentetrazol (PTZ) or low magnesium conditions (Chang et al., 2016; Figure 2) or in 4-aminopyridine induced *ex vivo* seizure models (Thio et al., 2000). Although the role of ketones in the ketogenic diet is controversial there is evidence in support of their efficacy. Ketones have been demonstrated to regulate mitochondrial function (Kim et al., 2015b) implicating cellular energy regulation. The ketogenic diet has also been suggested to function in seizure control through increasing activation of adenosine A1 receptors in a mouse model, however it remains to be determined if this effect is through ketone-dependent or fat-dependent mechanisms (Masino et al., 2011). Ketones have also been demonstrated to regulate synaptic KATP channels, providing a further potential mechanism in seizure control (Kim et al., 2015a; Li et al., 2017). Finally, ketones may function through epigenetic effects, regulating gene expression in relation to seizure susceptibility (Kobow et al., 2013; Lusardi et al., 2015). A comprehensive evaluation of the current experimental understanding of the efficacy of ketone bodies is reviewed elsewhere (Simeone et al., 2018).

The first investigation of medium-chain fatty acids relating to seizure control arose through several papers identifying elevated levels of both octanoic acid and decanoic acid in the plasma of patients on the MCT ketogenic diet (Haidukewych et al., 1982; Sills et al., 1986; Dean et al., 1989). Here, decanoic acid was shown at an average level of 157 μM (87–552 μM) and octanoic acid at 310 μM (104–859 μM). The relatively low number of patients assessed in these studies (up to 12 individuals) prevented a correlation between fatty acid levels and seizure control. Subsequently, an *in vivo* study of straight-chain fatty acids identified strong effects of long-chain fatty acids (e.g., palmitic acid containing 16 carbons) in a picrotoxin-induced seizure model in mice, with small but significant effects of decanoic acid in delaying the onset of clonic convulsions without an effect on survival time. The opposite effect was found for decanoic acid with subcutaneous PTZ induced seizures, where survival time was increased but clonic convulsions were not delayed (Nakamura et al., 1990). The small magnitude of the effect and the large variability of response did not provide clear support for a mechanism of decanoic acid in seizure control. More compelling evidence was provided by later studies.

It has been identified in *Dictyostelium* that medium-chain fatty acids regulate phosphoinositide signaling, in a similar but more potent mechanism to valproic acid (Chang et al., 2012). This study further demonstrated that some of these fatty acids blocked PTZ-induced epileptiform activity in an *in vitro* rat hippocampal model (Figure 2), again with a more potent effect than VPA. This anti-seizure effect occurred within 10 min of treatment, continually perfused with artificial CSF with high glucose content suggesting that the mechanism of seizure control was not dependent upon the build-up of ketones through the metabolism of fatty acids. Further studies identified that decanoic acid, and derivatives of octanoic acid show strong seizure control in a PTZ induced rat hippocampal model for seizure activity, again under perfusing CSF conditions unlikely to allow ketone generation (Chang et al., 2013). Medium-chain fatty acids were effective in a (perforant path stimulation-induced) status epilepticus *in vivo* model within 20 min of administration. A further study confirmed that branched-chain octanoic acid compounds showed strong structure-specific seizure control activity in a PTZ-induced hippocampal seizure model (Chang et al., 2015), in addition to blocking excitotoxic cell death induced by low magnesium levels in primary hippocampal neurons. Some related structures in this study showed potent control of epileptiform activity (Figure 2), without the negative side effect of VPA on histone deacetylase activity, widely associated with teratogenicity (Phiel et al., 2001; Gurvich et al., 2004).

Medium-chain fatty acids have also been demonstrated to function in seizure control in *in vivo* models. In one study in mice, seizure thresholds were increased in the *in vivo* 6 Hz model using a single bolus oral gavage dose of decanoic acid at 10 mmol/kg and 30 mmol/kg, and a similar increase was observed in the MES threshold model at 50 mmol/kg p.o., although no effect was observed at decanoic acid doses up to 50 mmol/kg p.o following seizure induction with i.v. PTZ (Wlaź et al., 2015). This group also showed that octanoic acid provided as a single bolus oral gavage dose at 20 mmol/kg and 30 mmol/kg significantly increased the dose of i.v. PTZ required to induce myoclonic twitch, and at 30 mmol/kg increased the dose of i.v. PTZ required to induce clonus, and that octanoic acid increased the seizure threshold above 10 mmol/kg in the 6-Hz model (Wlaź et al., 2012). The comparative importance of octanoic acid and decanoic acid was also examined in a mouse study. In this study, dietary treatment of mice with medium-chain triglycerides comprising either only octanoic acid or only decanoic acid was followed by induction of seizure like activity using both the 6 Hz model and the latency to first generalized seizure in the flurothyl model (Tan et al., 2016). This study showed that decanoic acid (only) triglycerides increased seizure thresholds whereas octanoic acid (only) triglycerides did not, supporting a role for decanoic acid in seizure control. It is also worthwhile to note that, since this study showed both decanoic acid and octanoic acid triglycerides provided a common level of ketosis, but only the decanoic acid triglyceride diet provided seizure control, this decanoic acid-dependent seizure control activity is likely to be unrelated to the generation of ketones. These studies therefore support a mechanism of seizure control through decanoic acid.

In many of these *in vivo* studies, a therapeutic role of fatty acids has been overlooked due to the perceived role of ketones as the mechanism of the diet. Therefore, it remains to be determined if free fatty acids may provide the therapeutic effects underlying the diet. For example, in one study (Mantis et al., 2014), augmentation of the ketogenic diet with glucose increased seizure susceptibility in a genetic mouse model, however, this study did not describe ketone levels or free fatty acid levels following glucose administration, thus it remains unclear if the role of glucose in these experiments was due to effects on ketosis or free fatty acid levels.

The MCT ketogenic diet leads to elevated levels of both octanoic acid and decanoic acid in the plasma of patients (Haidukewych et al., 1982), however, most studies suggest that decanoic acid and not octanoic acid is responsible for the therapeutic benefits of the diet (Chang et al., 2013, 2016; Hughes et al., 2014; Tan et al., 2016). So is there a benefit of including octanoic acid in the diet? Interestingly, recent studies have shown that octanoic acid, rather than decanoic acid, is preferentially metabolized in neurones by β-oxidation (Khabbush et al., 2017). This finding suggests that the presence of octanoic acid in the MCT ketogenic diet may allow decanoic acid to escape catabolism, thus accumulating, to enhance a therapeutic mechanism in preventing seizures. This finding supports an earlier *in vivo* study in mice using the 6 Hz seizure test that demonstrates an increased anticonvulsant activity of combined octanoic acid and decanoic acid in comparison to decanoic acid alone (Wlaź et al., 2015).

## Cellular Targets for Free Fatty Acids in Relation to Neuronal Excitability and Seizure Control

Two molecular mechanisms for decanoic acid have recently been proposed. As an acute mechanism for seizure control, decanoic acid has been shown to reduce excitatory postsynaptic currents (EPSCs) using whole cell patch clamp recordings from CA1 pyramidal neurons, likely through inhibition of excitatory AMPA receptors (Chang et al., 2016). By expressing distinct AMPA receptor subunits (GluA1, GluA2 and GluA3) in a *Xenopus* oocyte model, a direct inhibitory effect of decanoic acid against AMPA receptors was then confirmed, enabling detailed electrophysiological characterization (Chang et al., 2016). These studies showed that decanoic acid directly inhibits the two most abundant AMPA receptors subunit combinations found in the brain, with greatest potency against GluA2/3 (IC_50_ = 0.52 mM) and GluA1/2 (IC_50_ = 1.16 mM). This inhibitory effect was voltage-dependent, where potency against GluA2/3 receptors at −80 mV (IC_50_ of 1.11 mM) was elevated following depolarization to −40 mV (to IC_50_ of 0.43 mM), suggesting stronger inhibitory activity during prolonged seizure activity.

As a chronic mechanism of action, decanoic acid has also recently been demonstrated to activate the nuclear receptor, PPARγ, leading to increased mitochondrial proliferation (Hughes et al., 2014). Using cultured neuronal cells, decanoic acid but not octanoic acid was shown to trigger mitochondrial biogenesis and elevate the activity of the mitochondrial complex I. Since seizure activity is commonly found arising from a wide array of mitochondrial mutations (Zsurka and Kunz, 2015), this mechanism of decanoic acid is thought to increase ATP availability and improve brain energy metabolism, leading to an increase in seizure threshold and to a reduction in seizure activity following long term treatment.

## Conclusion

Understanding the mechanism of action of the MCT ketogenic diet in regulating neuronal excitability is critical for improving the treatment of patients with drug resistant epilepsy. Although the diet has clearly shown therapeutic relevance (Liu, 2008; Neal et al., 2008), evidence for a ketone-dependent mechanism in this function remains limited. The recent proposal for the efficacy of the diet is that fats provided through triglycerides in MCT supplements, in particular decanoic acid (Chang et al., 2013, 2015, 2016), may provide a direct function in blocking seizure activity independent of ketosis. Furthermore, novel compounds related to these medium-chain fatty acids may offer new approaches for seizure control without dietary restrictions (Chang et al., 2013). The studies outlined here provide a range of corollaries that should be considered in future experimentation. These include closely monitoring both fatty acid and ketone levels at a cellular and *in vivo* level, in both research and clinical settings to clarify the distinct contributions of fatty acids and ketones in epilepsy treatment. Furthermore, further studies may investigate improvements in the diet by modifying the fatty acid content of the diet, in addition to exploring other secondary targets of these fatty acids, and in the development of related chemicals that may function through the same therapeutic mechanism but lack the rapid metabolic degradation shown for medium-chain fatty acids.

## Author Contributions

EW (RHUL), MW (UCL) and RW (RHUL) contributed equally to the manuscript.

## Conflict of Interest Statement

The authors declare that the research was conducted in the absence of any commercial or financial relationships that could be construed as a potential conflict of interest.
